# Pattern variability in a computational model of respiratory rhythm generation

**DOI:** 10.1186/1471-2202-13-S1-P139

**Published:** 2012-07-16

**Authors:** Joonsue Lee, Chris Fietkiewicz

**Affiliations:** 1 Dept of Biomedical Engineering, Case Western Reserve University, Cleveland, OH, 44107, USA; 2 Dept of Electrical Engineering and Computer Science, Case Western Reserve Univ., Cleveland, OH, 44107, USA

## 

Variability is inherent to rhythmic physiological patterns. Fluctuations in neural patterns are often regarded as noise. However it has been suggested that neurally generated rhythms result from systems that are not homeostatic but instead are homeodynamic [1][2]. In this view, excessive variability may indicate an instability while insufficient variability could result from an inability to adapt to environmental stressors. Understanding the inherent mechanisms of variability in the nervous system may enable us to use statistical measures to evaluate stability and robustness.

Studies have addressed the presence of variability of the neural control of respiration in both the clinical context [3] as well as the experimental context [4]. Experimental studies have primarily focused on *in vivo* models that incorporate chemosensory and mechanosensory feedback which are likely to be necessary mechanisms of respiratory variability. A fundamental source of respiratory pattern generation is the preBötzinger complex (preBötC) which is part of the ventral respiratory column located in the brainstem. Here we use a computational model of the preBötC to analyze the variability of behavioral patterns at both the population and single-cell levels.

Our model is based on previous computational studies of the *in vitro* respiratory slice [5][6]. It utilizes Hodgking-Huxley style neurons [7] that are divided into four cell types: hypoglossal motoneurons, premotoneurons, and preBötC cells that are configured for bursting by means of a persistent Na^+^ current. The architecture consists a feedforward hierarchy such that motoneurons are driven by premotoneurons and premotoneurons are driven by preBötC cells. The preBötC cells receive inputs from each other and also receive random tonic drive in the form of excitatory and inhibitory postsynaptic currents. This tonic drive represents chemosensory and mechanosensory feedback. Hierarchical levels are randomly connected with a fixed connection probability or density. Additionally the model includes extracellular K^+^ diffusion/buffering and a cellular Na^+^/ K^+^ exchange pump. We analyzed the burst patterns of individual cells as well as the spike histogram of the hypoglossal motoneuron population which represents the whole-nerve activity recorded from cranial nerve XII in experiments. Variability was measured for both the single-cell bursts and motoneuron population bursts. Burst properties that were analyzed included respiratory cycle length, inspiratory phase length, and peak burst amplitude for the population spike histogram. Three variability measures were used: the linear measure of coefficient of variation and two nonlinear measures: mutual information and sample entropy.

We focused on two network areas and their influence on burst pattern variability. The first was the tonic drive to preBötC cells. We varied the quantity of tonic inputs and connection densities. We found that an increase in the connection density to preBötC cells produced a decrease in overall linear and nonlinear variability. This is due to the increase in postsynaptic event correlation that results from the combined common inputs. The second area that was studied was the interconnection density between the preBötC cells themselves. Again an increase in connection density produced a decrease in overall linear and nonlinear variability. However, this effect was not as pronounced as that of the tonic drive.
